# Juvenile idiopathic arthritis fibroblast-like synoviocytes influence chondrocytes to alter BMP antagonist expression demonstrating an interaction between the two prominent cell types involved in endochondral bone formation

**DOI:** 10.1186/s12969-020-00483-0

**Published:** 2020-11-16

**Authors:** Megan M. Simonds, Amanda R. Schlefman, Suzanne M. McCahan, Kathleen E. Sullivan, Carlos D. Rose, AnneMarie C. Brescia

**Affiliations:** 1 Nemours Biomedical Research, Nemours A.I. duPont Hospital for Children, 1701 Rockland Rd, Wilmington, DE 19803 USA; 2 Rheumatology, Nemours A.I. duPont Hospital for Children, Wilmington, DE USA; 3 Rheumatology, Johns Hopkins All Childrens, St. Petersburg, FL USA; 4 Allergy and Immunology, Children’s Hospital of Philadelphia, Philadelphia, PA USA

**Keywords:** Fibroblast, Synoviocyte, Chondrocyte, TGFβ, BMP4, BMP antagonists, Hypertrophy, Proliferation, Endochondral bone, Juvenile idiopathic arthritis

## Abstract

**Background:**

To examine critical interactions between juvenile idiopathic arthritis synovial fibroblasts (JFLS) and chondrocytes (Ch), and their role in bony overgrowth seen in patients with juvenile idiopathic arthritis (JIA).

**Methods:**

Control (CFLS) and JFLS were cultured in synoviocyte media containing recombinant BMP4. Ch were cultured in either CFLS or JFLS conditioned-media without stimulation. Media supernatants were analyzed by ELISA. RNA from conditioned media experiment was analyzed by ClariomS microarray.

**Results:**

As expected, genes expressed in untreated JFLS and CFLS cultured in synoviocyte media were similar to each other and this expression differed from untreated Ch cultured in chondrocyte media. JFLS favor BMP ligand gene expression while downregulating TGFβ receptors’ expression. Noggin and chordin, antagonists with high affinity for BMP4, are JFLS- but not Ch-preferred regulators of BMP signaling. Compared to Ch, JFLS overexpress collagen X (COLX), a marker of chondrocyte hypertrophy. Exogenous BMP4 causes JFLS to significantly decrease expression of noggin and collagen II (COL2), a marker of chondrocyte proliferation, and causes overexpression of COLX and alkaline-phosphatase (ALP). Chondrocytes cultured in JFLS-conditioned media (Ch-JFLS) express BMP genes and favor chordin protein expression over other antagonists. Ch-JFLS have significantly increased expression of COL2 and significantly decreased expression of COLX.

**Conclusions:**

These data suggest JFLS, in the presence of BMP4, undergo hypertrophy and that JFLS-conditioned media influence chondrocytes to become highly proliferative. To the authors’ knowledge, no prior study has shown that JFLS and chondrocytes play a direct role in the bony overgrowth in joints of patients with JIA and that BMPs or regulation of these growth factors influence the interaction between two prominent synovial cell types.

**Supplementary Information:**

The online version contains supplementary material available at 10.1186/s12969-020-00483-0.

## Background

The pathogenesis of Juvenile Idiopathic Arthritis (JIA) is not completely understood, but it is believed that fibroblast-like synoviocytes (FLS) play a critical role in disease progression and may contribute to joint growth disturbances in severe forms of JIA [1, 2]. FLS are one of the main cell types of the synovial lining and are considered to be key effector cells in the pathogenesis of adult rheumatoid arthritis (RA) [3]. FLS are known to contribute to cartilage destruction and joint damage in RA through production of cytokines, degradative enzymes, and inflammatory molecules [4–6]. While the interaction between FLS and inflammatory cells has been studied, their influence on growth plate chondrocytes has not been elucidated. There is significant interplay between FLS and chondrocytes, which has been studied primarily in RA models [7, 8]. Understanding how these two prominent cell types cooperate is necessary for revealing the mechanisms by which leg-length discrepancies and condylar hypertrophy occur in joints of patients with JIA.

We previously reported that JIA FLS (JFLS) have a chondrocyte-like phenotype and upregulated expression of Bone Morphogenetic Protein 4 (BMP4) [1], a finding that suggests increased chondrogenesis [9]. There is evidence from in vitro studies of animal models and RA [10] that suggest FLS derived from the pannus are classified as cartilage-like, having both phenotypic and functional characteristics similar to that of chondrocytes, consistent with our observations of FLS. The addition of chondrogenic growth factors, such as TGFβ or BMPs, promote proliferation and enhance chondrocyte differentiation in RA FLS signifying that they have been “primed” for that lineage commitment [11]. Although growth disturbances are more profound in JIA than RA, there have been no comparable studies of FLS from JIA patients. Dysregulated growth, epiphyseal deformity, and leg length discrepancy are common long term disabilities observed in JIA. Endochondral bone formation (EBF) is the process whereby chondrocytes proliferate, hypertrophy, then undergo apoptosis leaving behind extracellular matrix which forms the scaffolding for blood vessels, osteoclasts, bone marrow cells, and osteoblasts to invade and construct new bone [12]. We postulate that the chondrocyte-like phenotype observed in FLS may contribute to growth abnormalities in JIA.

TGFβ and BMPs are important members of the TGFβ superfamily and are widely known to contribute to cartilage and bone formation. BMPs promote proliferation and differentiation in chondrocytes (Ch) [13]. Levels of BMPs are elevated in the synovium and synovial fluid of patients with RA while phosphorylation of SMAD1/5/8 is increased in RA synovial biopsies, indicating BMP activation persists despite inflammation control with treatment [14–17]. Smad2/3 and Smad1/5/8 respond to activated TGFβ and BMP receptors, respectively, by forming complexes with Smad4 and translocating to the nucleus to regulate target gene transcription [18, 19]. SMURF2 and Smad7 are intracellular regulators of TGFβ signaling [19]. BMP1 is a secreted metalloprotease that inhibits chordin, which itself is a negative regulator of BMP signaling [20].

In this study, we examined how BMP4 influences the pluripotency of FLS and the interaction of FLS and chondrocytes in vitro*.* It has been well-established that BMPs are essential for chondrocyte terminal differentiation and play a central role in EBF [21, 22]. Because FLS have a chondrogenic phenotype in JIA that is similar to that in RA, we exposed JFLS to exogenous BMP4. Furthermore, because the regulation of BMPs through antagonists can promote FLS differentiation toward hypertrophy, we set out to determine how BMP4 affects expression of BMP antagonists in FLS and explore the effect of FLS-conditioned media on chondrocytes. We aimed to establish a potentially novel finding with clinical impact for the direct contribution of FLS to dysregulated growth seen in joints from patients with JIA.

## Methods

### Selection of samples

Synovial fluid samples were obtained from our Institutional Review Board-approved repository. Patients who underwent clinically indicated arthrocentesis were offered inclusion into the repository and informed consent was obtained. As part of the arthrocentesis procedure, synovial fluid (SF) is aspirated from the affected joint prior to steroid injection. We selected three subjects from the repository with persistent oligoarticular JIA, as defined by the International League of Associations for Rheumatology (ILAR) because this is the least severe subtype of JIA with minimal interventions. Inclusion criteria is as follows: no prior steroid injections, patients were taking nonsteroidal anti-inflammatory drugs (NSAIDs) only, and samples remain persistent for at least 1 year without extending. Exclusion criteria is as follows: medication restrictions include methotrexate and biologics, extended JIA, polyarticular JIA, undifferentiated JIA, Psoriatic JIA, ERA subtype JIA, IBD-related arthropathy, HLA B27+, Syndromic arthropathy (such as associated with Down syndrome), HIV-related arthropathy, Lyme arthritis, Thyroiditis-associated arthropathy. We procured three normal human chondrocyte cell lines (402 K-05a) and three normal human FLS cell lines from Cell Applications, Inc. (408 K-05a). All purchased cell lines were from individual donors.

### Cell culture

Cells were isolated from SF obtained from arthrocentesis, cultured in DMEM with 15% FBS at 37C with 5% CO2 in T-75 flasks and harvested at passage 3 for experiments, as this allows for an adhesive monolayer of synovial fibroblasts and eliminates inflammatory cells from culture. Three biological replicates of JFLS cell lines cultured from synovial fluid samples and three biological replicates of CFLS cell line samples were plated in their respective media after three passages. Approximately 2.0 × 10^6 cells per patient sample were trypsinized, centrifuged for 5 min at 500 rpm, and resuspended in appropriate media. Cells were then plated in 6-well plates at 3 ml/well. For treated samples, cell lines were exposed to 1000 ng/ml of recombinant human BMP4 (R&D Systems) in DMEM medium that contained 15% FBS. At 2, 6, 12, 18, and 24 h after plating, media supernatant samples were collected for untreated cells and cells treated with exogenous BMP4.

### FLS-conditioned media

To obtain conditioned media, three biological replicates of CFLS lines and three biological replicates of JFLS lines were grown to confluence. Media was replaced with fresh and cell lines were incubated an additional 48 H*. media* from all three CFLS lines were pooled and used as CFLS conditioned media, and media from the three JFLS lines were pooled and used as JFLS conditioned media. Three biological replicates of Ch lines were grown until confluence, then washed with PBS, trypsinized, and resuspended in either the pooled CFLS conditioned media (Ch-CFLS), pooled JFLS conditioned media (Ch-JFLS), or normal Ch growth medium as a control. Pooled media allowed for us to take into account variation between patients and components in SF. Specifically, both FLS and Ch were cultured in T-75 flasks at 37C and 5% CO2 in their respective media (FLS growth medium, Cell Applications, Inc. 415–500 and chondrocyte growth medium, Cell Applications, Inc. 411–500). At confluence, there were approximately 2.0 X10^6 cells per cell line. For subculturing, cells were trypsinized and centrifuged for 5 min at 500 rpm. Chondrocytes were resuspended evenly in conditioned media from either CFLS or JFLS and 3mls of cell suspension were plated in each well of 6-well plates. Cell culture supernatants were collected at 6, 12 and 24 h after exposure to conditioned media.

### Enzyme-linked Immunosorbent assay

Every ELISA was performed using 3 biological replicates from untreated Ch, CFLS, JFLS, as well as, CFLS and JFLS exposed to exogenous BMP4, and Ch cultured in either CFLS or JFLS-conditioned media and plated in triplicate. Protein concentrations from cell culture supernatants were measured using Bradford assay. Each well for each 96-well plate contained equal amounts of protein depending on detection recommendations from manufacturer’s protocols for all ELISAs. Specifically, at the appropriate timepoints, 1 ml of cell culture media supernatant was collected. Protein concentration in this aliquot was determined by Bradford assay. Total protein was then adequately diluted to 100 ng/ml and ELISA were plated so that each well contained the same amount of protein/volume based on the manufacturer’s protocols.

ELISA kits from LifeSpan Biosciences, Inc. were used to detect gremlin (LS-F21084), noggin (LS-F24239), collagen II (LS-F26824), and collagen X (LS-F13131). ELISA kits from Raybiotech Inc. were used to detect follistatin (ELH-FOLLISTATIN) and chordin (ELH-CHRDL). Alkaline Phosphatase Assay Kit was purchased from Abcam Inc. and performed according to manufacturer’s protocol (ab83369).

### GeneChip whole transcriptome expression analysis

Arrays were processed following the standard Affymetrix protocol [23]. Gene expression was determined using the SST-RMA algorithm in Expression Console (Affymetrix).

### Data analysis

RankProduct analysis was performed using the R package, RankProd [24, 25] to identify differentially expressed genes between different cell types with estimated percentage false prediction (pfp) < 0.01 considered as significant. LIMMA analysis was performed using the R package, limma [26] to determine differentially expressed genes between chondrocytes cultured in different conditions with 5% FDR considered as significant. Ratios comparing 24 h to 6 h time points were calculated using linear intensity for each sample for Ch, CFLS, JFLS, Ch-CFLS, and Ch-JFLS. Changes over time were graphed, and t-tests were used to determined statistical significance. For ELISA on BMP4 treated samples, equal amounts of total protein were plated for each biological replicate and those replicates were plated in triplicate. Optical density readings were converted to pg/ml using standard curves and normalized to the zero time point allowing for correction between triplicates and slight variations in protein concentrations due to pipetting. Ratios were calculated at each time point by dividing normalized treated concentrations by normalized untreated concentrations. T-tests were used to determine statistical significance between untreated and treated for each cell type. For Ch cultured in FLS-conditioned media, equal amounts of total protein were plated for each biological replicate and those replicates were plated in triplicate. Optical density readings were converted to pg/ml using standard curves and ratios were calculated by dividing the protein concentration at 24 h by the protein concentration at the 6 h time point to establish fold-change over time. T-tests were used to determine the difference between Ch and Ch cultured in CFLS-conditioned media, as well as Ch and Ch cultured in JFLS-conditioned media.

## Results

### Gene expression analysis of Ch, JFLS and CFLS

**Fig. 1:**
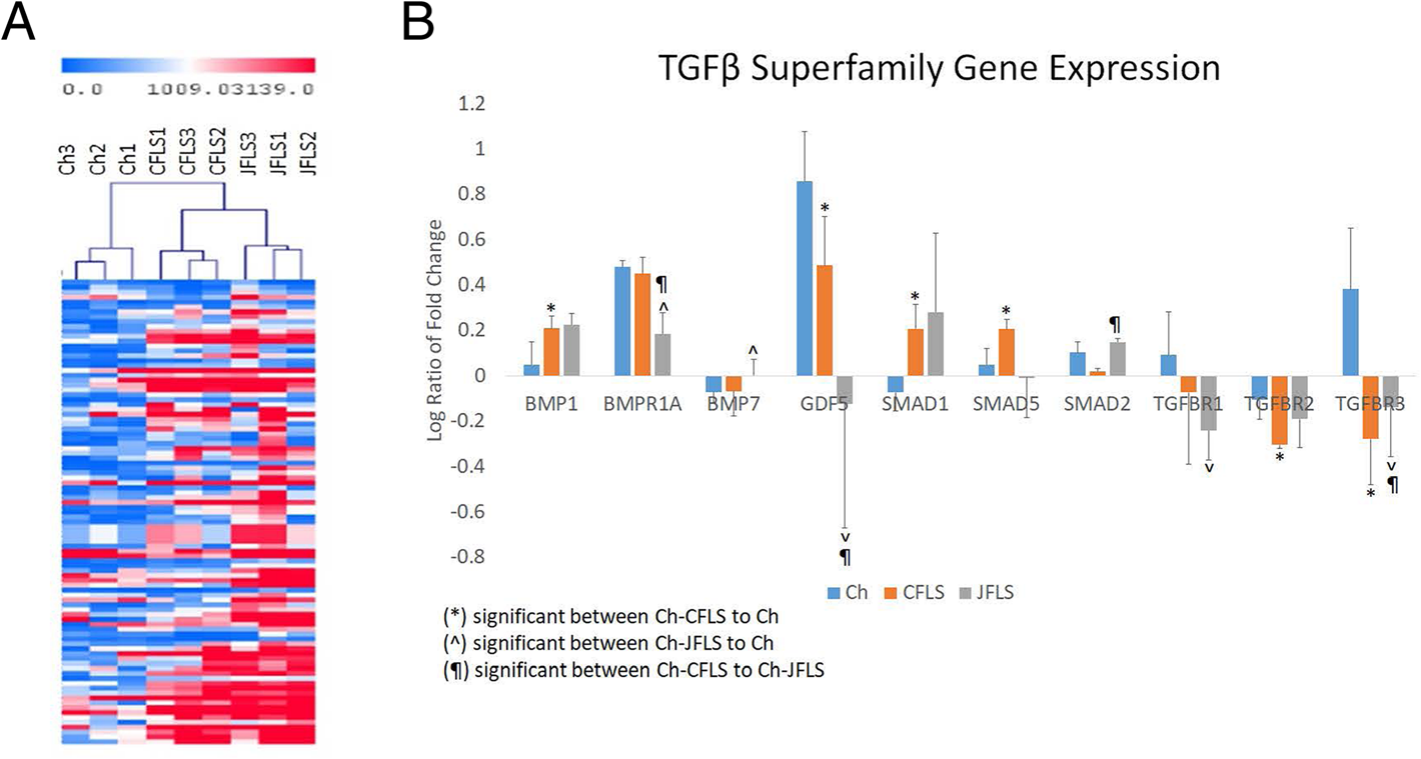
Unsupervised hierarchal clustering comparing chondrocytes (Ch) and fibroblast-like synoviocytes (FLS) after 6 h in culture and differentially expressed genes specific to TGFβ and BMP signaling in these cells. **a**, Unsupervised hierarchical clustering of 104 differentially expressed genes (estimated percentage false prediction (pfp < 0.01, Rank Product) JFLS have similar gene expression patterns to CFLS and differ from Ch. **b**, Using a manually curated list of 27 genes specific to these signaling pathways, we examine changes in gene expression over 24 h using linear expression values. CFLS have significantly higher levels of BMP signaling via the activation of SMAD1/5 while JFLS overexpress BMP ligand, BMP7 compared to Ch (*p* < 0.05, t-test). Concurrently, there is a reduction in the gene expression of TGFβ receptors in CFLS and JFLS (TGFBR2/TGFBR3 and TGFBR1/TGFBR3, respectively) when compared to Ch (*p* < 0.05)

To best understand the biology of FLS, we utilized an unbiased approach to globally characterize commonalities and discordances between the cell types via microarray. Rank Product analysis revealed 104 genes differentially expressed in at least one comparison (Ch vs CFLS, Ch vs JFLS, CFLS vs JFLS) after 6 h in culture (pfp < 0.01) (Supplemental Table 1). We generated an unsupervised hierarchical clustering of Ch and FLS after 6 h in culture using these 104 genes (Fig. 1a). JFLS overall have a gene expression pattern similar to that of CFLS and with features that are different from chondrocytes. Given that TGFβ and BMP contribute to the pathogenesis of RA, and BMPs generally regulate bone development [1, 27–29], using Ingenuity Pathway Analysis (IPA) we manually curated a list of 27 genes related to TGFβ/BMP signaling including Smads, BMP ligands and receptors, and TGFβ ligands and receptors (Supplemental Table 2). FLS in culture had significantly higher expression levels of genes related to BMP signaling (BMP1, Smad1, Smad5 in CFLS) (BMP ligand BMP7 in JFLS) when compared to Ch (Fig. 1b). Both CFLS and JFLS in culture had significantly lower expression levels of TGFβ receptors (TGFBR2/TGFBR3 and TGFBR1/TGFBR3, respectively) when compared to Ch (Fig. 1b). Thus, a major difference in FLS compared to chondrocytes was demonstrated by a significant increase in BMP signaling gene expression as compared to TGFβ signaling, supporting our previous studies that JFLS robustly express BMP ligands.

### BMP antagonists in JFLS

**Fig. 2:**
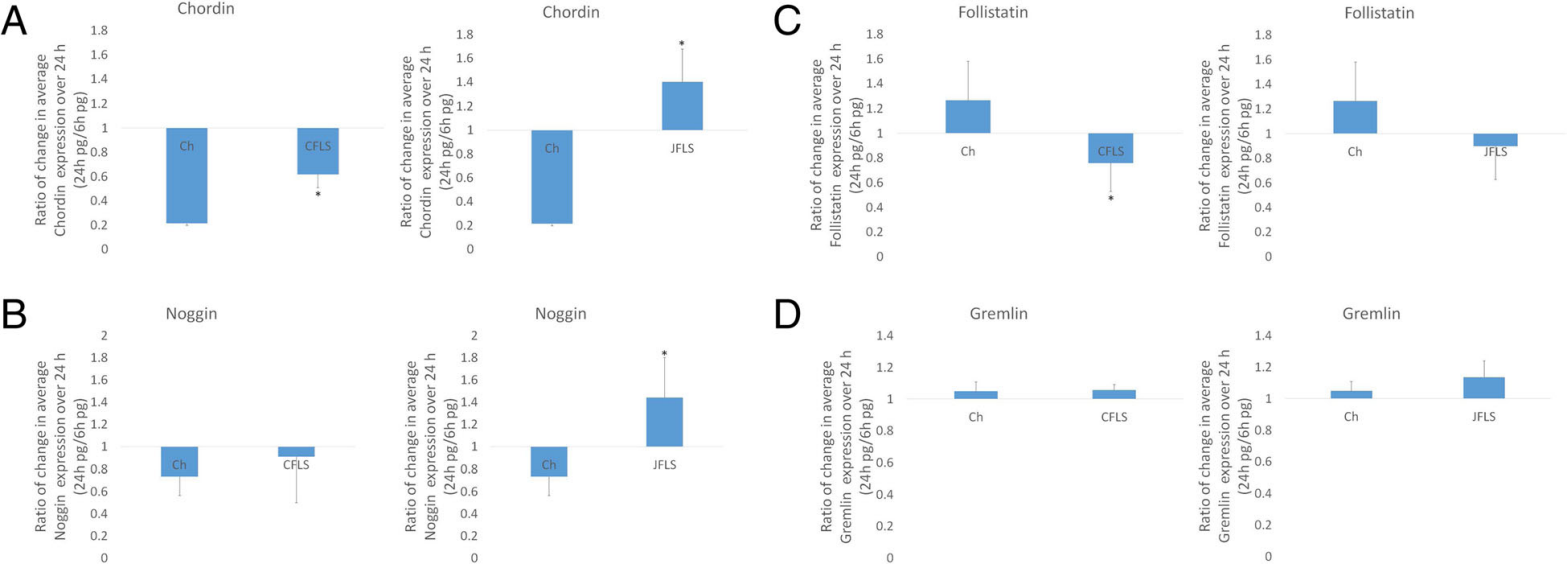
Protein expression of BMP antagonists: chordin, noggin, follistatin, and gremlin. JFLS significantly overexpress chordin (**a**) and noggin (**b**) when compared to Ch (*p* < 0.05, t-test) as determined by ELISA on cell media supernatants. CFLS exhibit a significant decrease in follistatin when compared to untreated Ch (**c**) while gremlin remained unchanged in FLS when compared to Ch (**d**)

Both types of FLS exhibited increased BMP ligand expression compared to chondrocytes. BMPs are central to chondrocyte differentiation into bone [23], and their biological effect is strongly modulated in vivo by the balance of antagonists. We used ELISA to define the production of antagonists in CFLS and JFLS compared to Ch. JFLS expressed high levels of chordin and noggin when compared to both CFLS and Ch (Fig. 2a and b). While JFLS and CFLS produce similar levels of BMPs, the expression of the antagonists differs between the two cell types and affects the balance of BMP effect.

### JFLS have features of chondrocytes

**Fig. 3:**
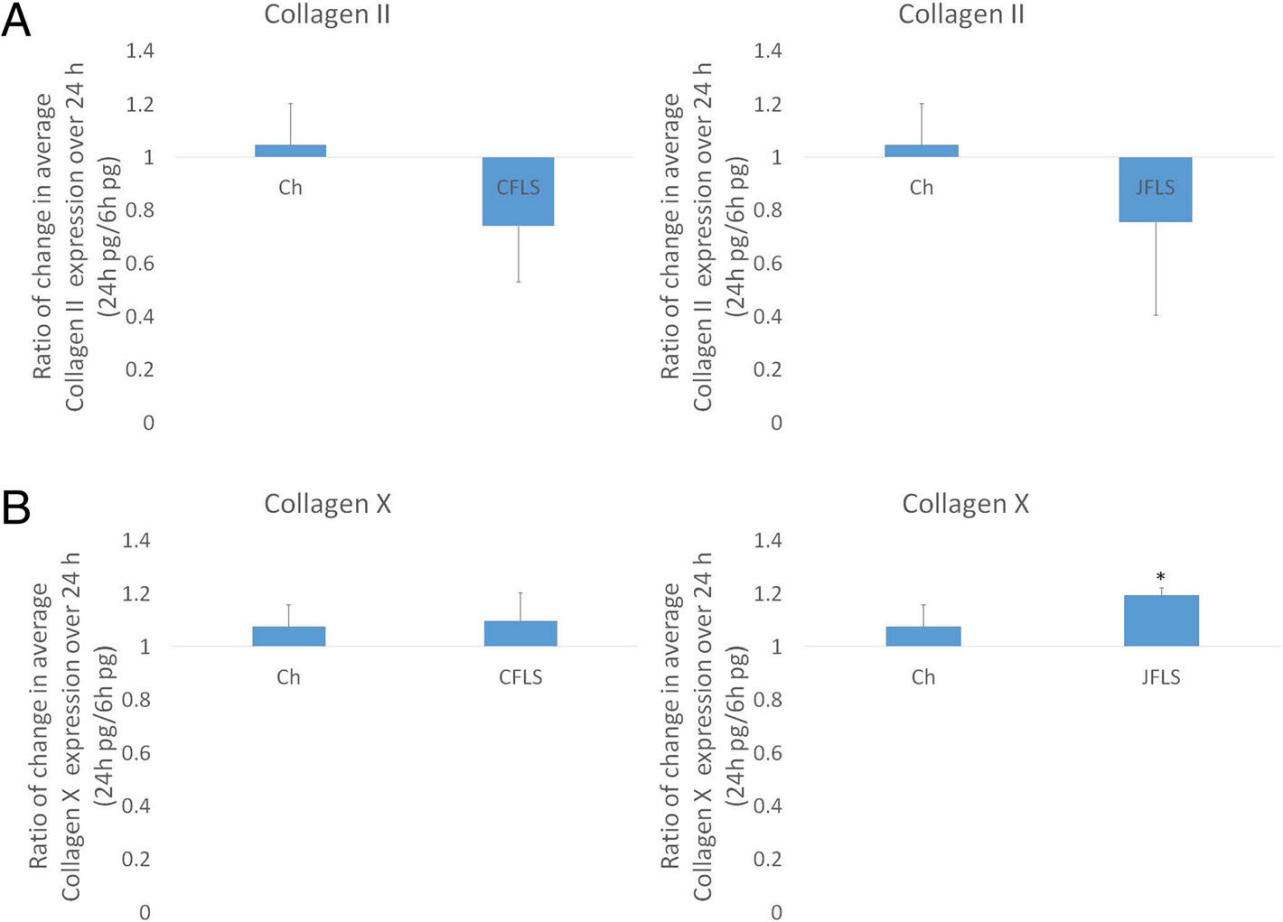
Protein expression of collagen II (COL2), a marker of proliferating chondrocytes and collagen X (COLX), a marker of chondrocyte hypertrophy as determined by ELISA on cell media supernatants from FLS and Ch. There were no significant changes in COL2 expression between FLS and Ch (**a**). COLX was significantly overexpressed in JFLS compared to Ch (*p* < 0.05, t-test) (**b**)

We previously reported that JFLS have a chondrocyte-like phenotype [1]. We examined expression of downstream proteins specific to chondrocyte differentiation. We measured expression of markers of proliferating chondrocytes (Collagen II) and hypertrophic chondrocytes (Collagen X) using ELISA (Fig. 3a and b). Collagen X (COLX) was significantly upregulated in JFLS compared to Ch, suggesting that JFLS in vitro express markers of late-stage chondrocyte differentiation (Fig. 3b). Since there was no significant difference between Ch and CFLS when measuring COLX, these findings suggest JFLS have characteristics of hypertrophic chondrocytes and further distinguish the JFLS from CFLS when compared to Ch.

### Exogenous BMP4 regulates BMP antagonists

**Fig. 4:**
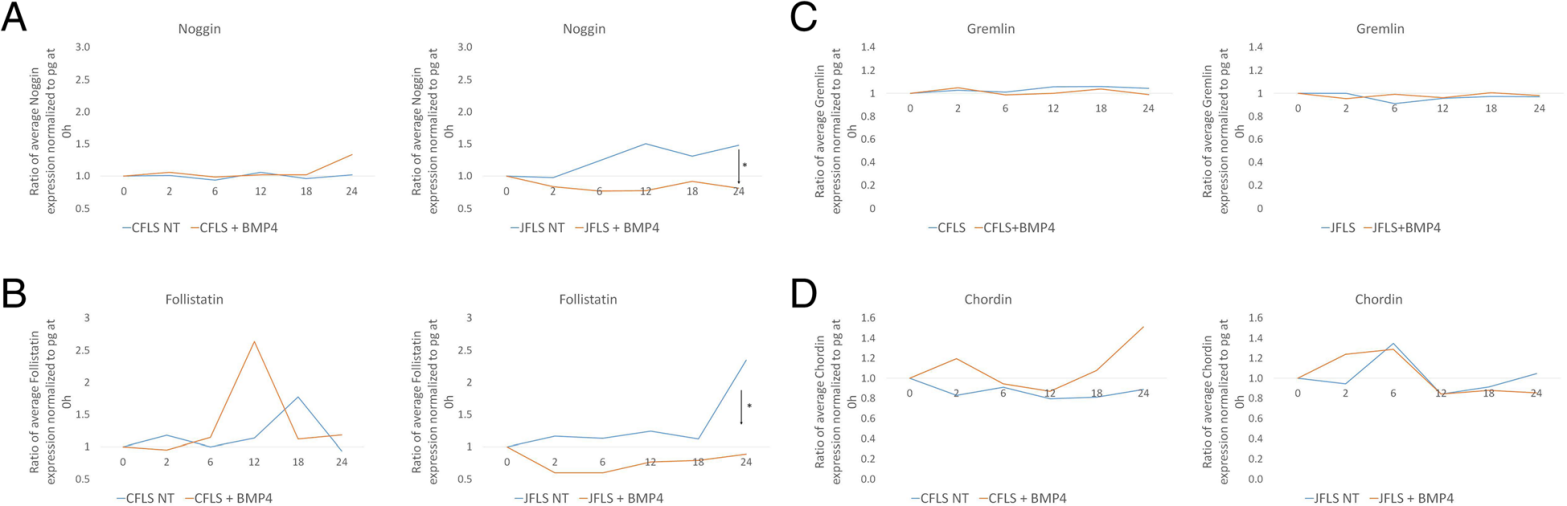
Using ELISA to study the effects of exogenous BMP4 on protein expression of BMP antagonists in cell media supernatants over 24 h. JFLS, in the presence of BMP4, significantly decrease expression of noggin and follistatin (**a** and **b**) (*p* < 0.05) while gremlin and chordin remained unchanged in FLS exposed to exogenous BMP4 (**c** and **d**)

Although CFLS and JFLS expressed BMPs comparably they showed differential expression of BMP antagonists. The effects of exogenous BMP4 on expression profiles was investigated since BMP4 is a known regulator of chondrogenesis [30]. We exposed FLS to recombinant BMP4 in vitro and studied the effects on expression of BMP antagonists. CFLS and JFLS cells were cultured in media with the addition of recombinant BMP4 for 24 h (Half-life < 24 h). CFLS exhibited little change in expression of antagonists over 24 h (Fig. 4). In contrast, the effect of BMP4 on JFLS in cell culture was characterized by significant decreases in noggin and follistatin, two BMP4 antagonists with high affinities for BMP4, demonstrating an aberrant response to BMP4 by JFLS (Fig. 4a and b).

### Exogenous BMP4 regulates collagen expression

**Fig. 5:**
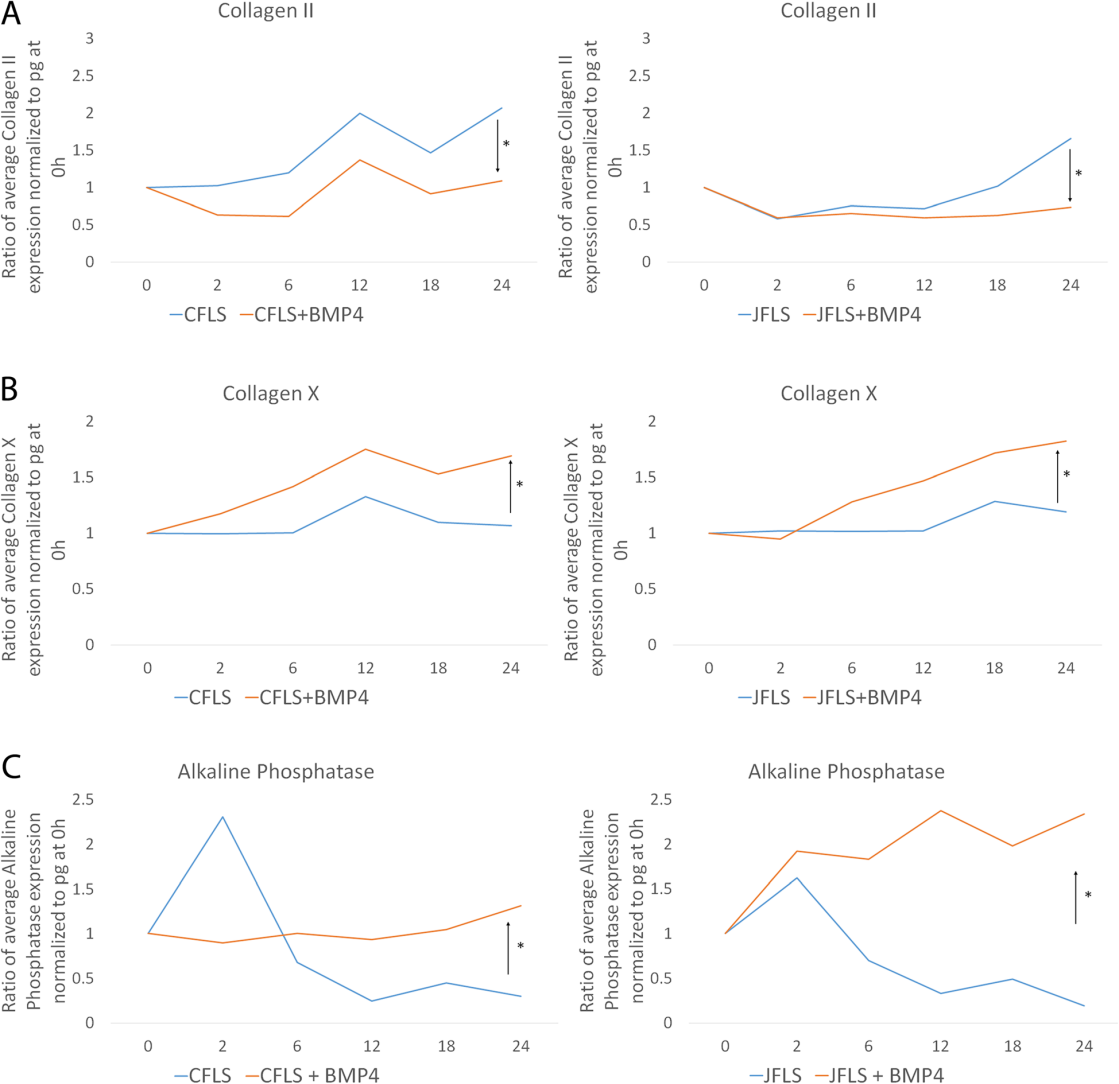
Using ELISA to study the effects of exogenous BMP4 on protein expression of markers of chondrocyte differentiation. Exogenous BMP4 causes a significant decrease in COL2 in JFLS (**a**) (*p* < 0.05) and an increase in COLX in both CFLS and JFLS (B) (*p* < 0.05). CFLS and JFLS, in the presence of BMP4, overexpress bone-derived alkaline phosphatase (ALP), a marker secreted by bone cells and hypertrophic chondrocytes (**c**) (*p* < 0.05)

JFLS were shown to express higher levels of COLX than CFLS. We hypothesized that COLX expression could be regulated by BMP4 in FLS. Collagen II (COL2) and COLX expression was measured after treatment with BMP4, as above, using ELISA. COL2 significantly decreased over time in CFLS and JFLS compared to untreated cells (Fig. 5a) while COLX significantly increased over 24 h in both CFLS and JFLS compared to untreated FLS (Fig. 5b). Thus, both types of FLS respond to BMP4 by increasing expression of COLX, a hypertrophic chondrocyte marker.

### FLS express bone-derived alkaline phosphatase after BMP4 stimulation

To determine if JFLS have other features of chondrocyte lineage, we measured bone-derived alkaline phosphatase (ALP) in FLS exposed to exogenous BMP4. After 24 h in culture, CFLS and JFLS stimulated with BMP4 have significantly higher ratios of bone-derived ALP expression than untreated CFLS and JFLS (Fig. 5c). Notably, JFLS express significantly more bone-derived ALP than CFLS when both were treated with BMP4. Collectively, these data support our hypothesis that JFLS, in the presence of BMP4, acquire an abnormal chondrocyte-like protein expression signature. This finding may be important in the mechanism of bony overgrowth in patients with JIA.

### FLS have a direct influence on chondrocytes

**Fig. 6:**
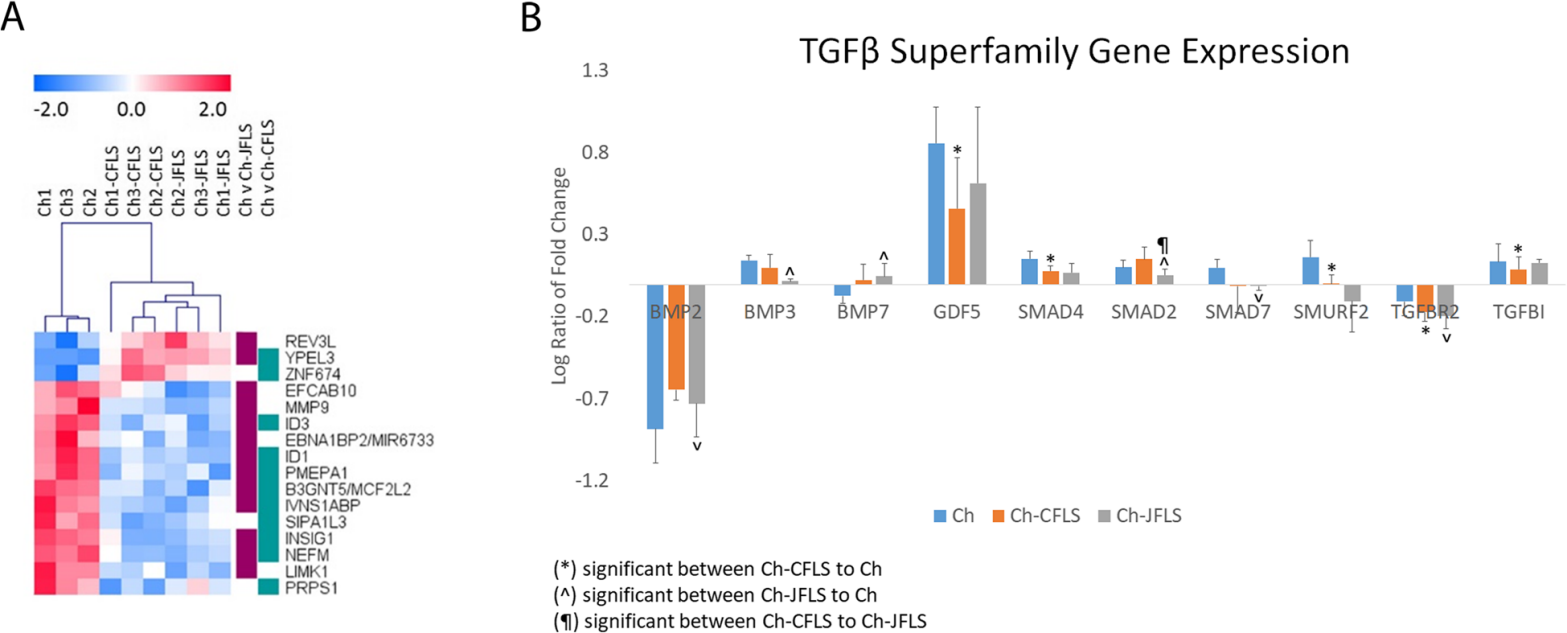
Unsupervised hierarchal clustering comparing chondrocytes cultured in FLS-conditioned media and untreated chondrocytes and differentially expressed genes specific to TGFβ and BMP signaling in these chondrocytes. LIMMA revealed 11 differentially expressed genes with a 5% FDR when comparing Ch and Ch-CFLS (Fig. 4**a**, teal bar) and 13 differentially expressed genes between Ch and Ch-JFLS (Fig. 4**a**, purple bar) after 6 h of exposure to conditioned media. Overlapping genes revealed those regulated by TGFβ/BMP (ID1, ID3, and PMEPA1). Analyzed changes in gene expression between untreated Ch compared to Ch-CFLS and Ch-JFLS over 24 h using the curated list of genes involved in TGFβ/BMP signaling (**b**). Ch-CFLS and Ch-JFLS had significantly lower expression levels of TGFβ-related genes when compared to untreated Ch (Ch-CFLS: SMAD4, SMURF2, TGFBR2, TGFBI and Ch-JFLS: SMAD2, SMAD7, TGFBR2) (*p* < 0.05). Ch-JFLS had significantly increased expression of BMP2 and BMP7 compared to untreated Ch (*p* < 0.05)

The interaction between synoviocytes and chondrocytes is thought to play an essential role in disease pathology of RA, which prompted us to investigate their relationship in JIA. Thus far, we have examined the effect of a single mediator, BMP4, known to be expressed by FLS, on the FLS themselves. To study the interplay between cell types using a model that more closely mimics JIA biology, we examined the gene expression of chondrocytes cultured in CFLS-conditioned media (Ch-CFLS) and chondrocytes cultured in JFLS-conditioned media (Ch-JFLS) using microarray. The addition of FLS-conditioned media altered the gene expression of Ch in culture. Eleven differentially expressed genes (DEG) were identified comparing Ch and Ch-CFLS (Fig. 6a, teal bar) and 13 DEG between Ch and Ch-JFLS (Fig. 6a, purple bar) after 6 h of exposure to conditioned media (5% FDR, LIMMA).

There were 8 genes that overlapped between the two comparisons, including three genes regulated by TGFβ/BMP: ID1, ID3, and PMEPA1. We analyzed the 16 DEG using Ingenuity Pathway Analysis (IPA) to assess the functional relevance. The top canonical pathways were (1) mouse embryonic stem cell pluripotency (2) unfolded protein response and (3) TGFβ signaling. The ID1 gene, which was downregulated in both Ch-CFLS and Ch-JFLS when compared to untreated Ch, had the highest fold change in the dataset. ID proteins are inhibitors of differentiation [31], suggesting that FLS could induce de-differentiation in chondrocytes through a TGFβ/BMP dependent mechanism.

### FLS-conditioned media downregulates TGFβ pathway genes and upregulates BMP pathway genes

Our microarray gene expression studies suggested a role for the TGFβ pathway. We therefore analyzed the change in gene expression of untreated Ch vs. Ch-CFLS and Ch-JFLS over 24 h using a curated list of genes involved in TGFβ/BMP signaling. Both Ch-CFLS and Ch-JFLS exhibited perturbations to the TGFβ gene set, although with effects specific for each type of conditioned media. Ch-CFLS had significantly lower expression levels of SMAD4, SMURF2, TGFBR2, TGFBI while Ch-JFLS had a significant decrease in SMAD2, SMAD7 and TGFBR2 (*p* < 0.05, t-test) (Fig. 6b). Additionally, Ch-JFLS had significantly increased expression of BMP ligands (BMP2, BMP7) when compared to untreated Ch (*p* < 0.05, t-test) (Fig. 6b). FLS conditioned media globally influenced chondrocytes to decrease TGFβ and increase BMP pathway molecules. Specific genes affected were both shared and distinct between Ch-CFLS and Ch-JFLS.

### BMP antagonists are regulated by FLS-conditioned media

**Fig. 7:**
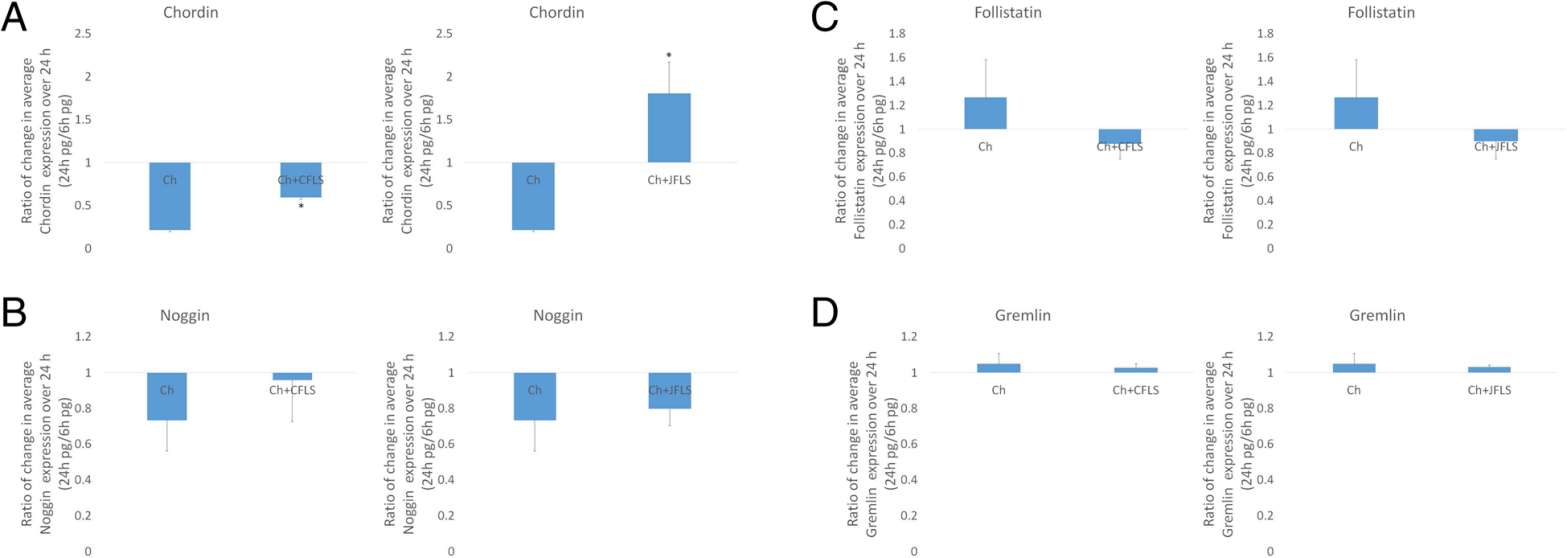
Protein expression of BMP antagonists: chordin, noggin, follistatin, and gremlin. Using ELISA to measure protein expression, both Ch-CFLS and Ch-JFLS significantly overexpress chordin when compared to untreated Ch with the greater difference seen in Ch cultured in JFLS-conditioned media (**a**) (*p* < 0.05) while noggin, follistatin, and gremlin remained unchanged (**b**, **c**, and **d**)

Because chondrocytes cultured in FLS-conditioned media upregulated BMP pathway genes, we evaluated the expression of BMP antagonists using this conditioned media model system. When Ch were cultured in FLS-conditioned media, chordin was the only antagonist that was discordantly affected by JFLS compared to CFLS conditioned media (Fig. 7a). The FLS conditioned media from both CFLS and JFLS led to modestly altered levels of noggin, follistatin, and gremlin. This discordant expression of chordin was also seen between CFLS and JFLS.

### FLS-conditioned media alters collagen expression

**Fig. 8:**
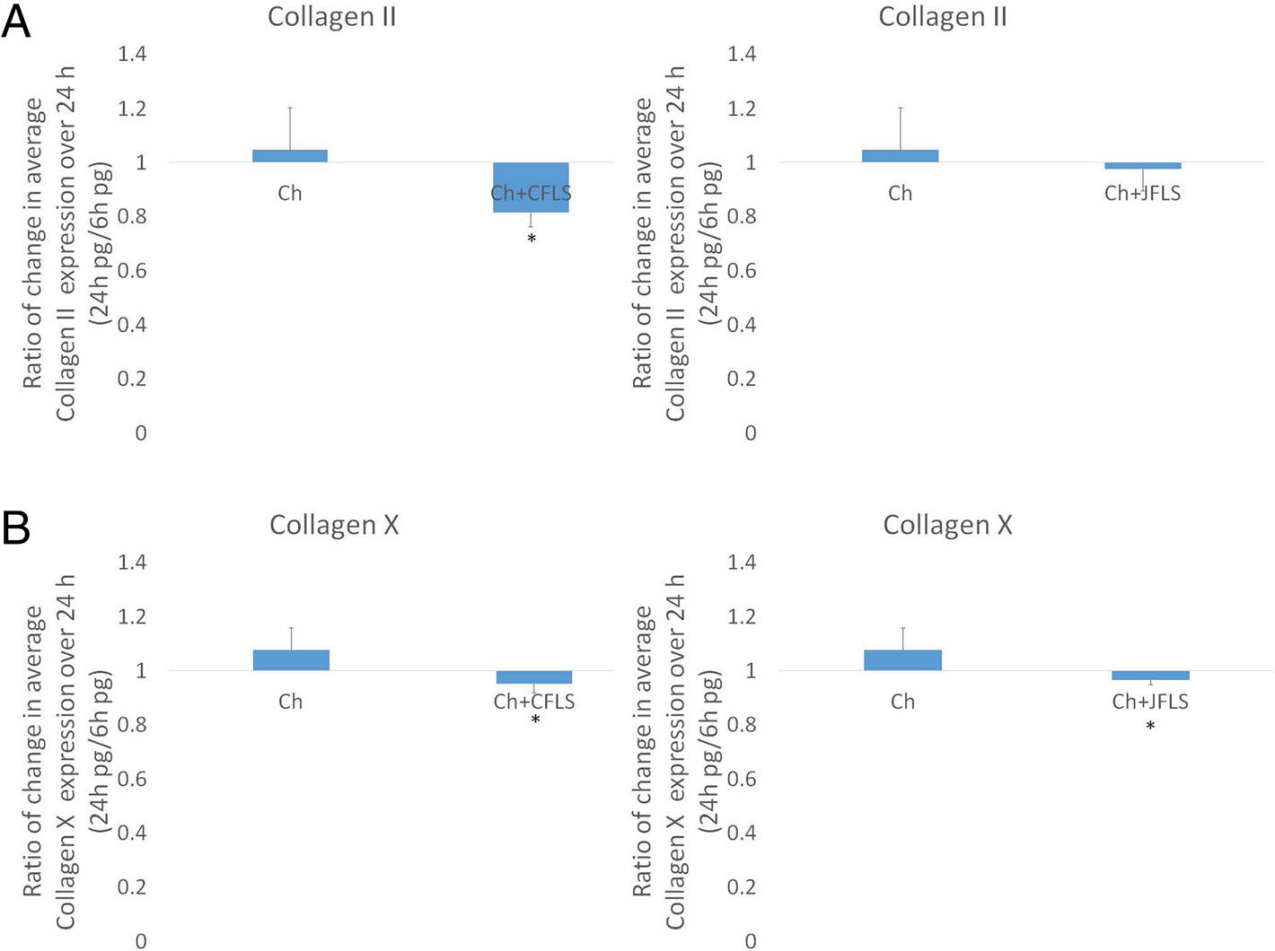
Protein expression of collagen II (COL2), a marker of proliferating chondrocytes and collagen X (COLX), a marker of chondrocyte hypertrophy as determined by ELISA on cell media supernatants. While both Ch-CFLS and Ch-JFLS decrease expression of proliferation marker, COL2, the difference was only significant in Ch cultured in CFLS-conditioned media (**a**). Hypertrophic marker, COLX is significantly downregulated in both Ch-CFLS and Ch-JFLS (**b**) (*p* < 0.05, t-test)

Cultured JFLS overexpress hypertrophic chondrocyte marker, COLX. This finding prompted us to analyze how FLS influence the expression of these protein markers in chondrocytes. The two collagen markers had decreased expression after treatment with conditioned media with the main difference being lower expression of COLX in Ch-CFLS and Ch-JFLS when each condition was compared to untreated Ch (Fig. 8b). Thus chondrocytes are substantively altered by exposure to ligands produced by FLS, indicating significant interactions that impact biology in the joint space.

## Discussion

Fibroblast-like synoviocytes (FLS) play a key role in the pathogenesis of RA [2] and may be as influential in JIA. These cells, in conjunction with chondrocytes, likely contribute to bony overgrowth in affected joints. This type of growth disturbance is thought to occur through endochondral bone formation, a process by which hypertrophic chondrocytes undergo apoptosis and provide the scaffolding for new bone invasion [12]. In avian limbs, BMPs drive chondrogenesis and in mammals, the BMP signaling pathway is involved in skeleton formation [32, 33]. In the canonical signaling pathway, BMPs are released into the extracellular space and bind to serine-threonine kinase receptors, leading to nuclear translocation of Smad 1/5/8 proteins, which regulate target gene expression. BMPs are also regulated by their antagonists which are highly redundant and critical to the final bone structure. BMP antagonists bind BMP proteins specifically, preventing them from interacting with their receptors. Chordin is expressed specifically in growth plate chondrocytes, articular chondrocytes, and osteoblasts and its expression is inversely related to chondrocyte maturation in developmental systems [34]. Chordin is an effective negative regulator of endochondral ossification. Ectopic expression of chordin in developing chick limbs attenuates chondrocyte hypertrophy in vivo [34] and addition of chordin to osteoarthritic chondrocytes in vitro showed a delay in cell maturation and hypertrophy [35]. Great advances have been made in the understanding of BMP biology and the role of the antagonists, yet there are few studies examining their roles in arthritis. This study brings together a rigorous examination of FLS from patients with JIA and uses a novel methodology to examine interactions of chondrocytes and FLS to probe the possible communications occurring in the joint space.

Synovial fibroblasts produce cartilage oligomeric matrix protein (COMP), and glycosaminoglycans similar to chondrocytes [36, 37]. We previously reported that JIA FLS have a chondrocyte-like phenotype based on mRNA and protein expression of cartilaginous markers including COL2, COMP, aggrecan (ACAN) and COLX [1]. In this study, we compared the genomic expression of chondrocytes, control FLS, and JIA FLS in vitro*.* There were more similarities than differences in gene expression related to TGFβ /BMP signaling between FLS lines and chondrocytes. BMP associated genes had significantly increased expression over TGFβ gene expression in FLS compared to Ch.

BMPs are potent inducers of endochondral bone formation, and their role has been studied in adult patients with ankylosing spondylitis [38, 39]. We posit that BMPs play a similar role in JIA regarding growth disturbance and leg-length discrepancies. Both normal and diseased FLS favor chordin, a potent inhibitor of BMP2, BMP4, and BMP7, over follistatin, gremlin, or noggin when compared to Ch. JFLS also demonstrated increased noggin expression while CFLS had lower expression of follistatin when compared to Ch. The absence of follistatin can cause skeletal abnormalities during development, specifically endochondral bone growth [40]. Noggin levels in conjunction with increased BMP4 allow for proper skeletal formation during development and inhibition of noggin leads to dysregulated endochondral ossification [41]. These data suggest Ch and FLS have compensatory mechanisms for balancing BMP signaling.

Due to the dysregulation of TGFβ/BMP signaling in FLS and protein expression of BMP antagonists, exogenous BMP4 was added to CFLS and JFLS in vitro. BMP isoforms including BMP2, BMP4, BMP5, and BMP7 are expressed in the perichondrium and are strong stimulators of chondrocyte maturation [34] and BMP4 promotes cartilage growth and chondrocyte proliferation [9]. In vitro, BMP4 causes the fibroblast cell line NIH/3 T3 to successfully differentiate towards chondrocyte lineage [42]. Exposing JFLS to exogenous BMP4 in culture resulted in an increase in BMP-related genes as opposed to genes specific to TGFβ signaling. With the addition of exogenous BMP4, FLS shifted from proliferative to hypertrophic chondrocyte-like phenotype with reduction in COL2 and increase in COLX, consistent with hypertrophic chondrocytes [43] and what has been described as the chondrogenic potential of RA FLS [44]. JFLS exposed to BMP4 expressed significantly higher levels of alkaline phosphatase (ALP) than both untreated JFLS and CFLS exposed to BMP4. It is suggested that high levels of serum ALP contributes to disease progression in patients with RA [45, 46].

During the process of endochondral bone formation, chondrocytes proliferate, hypertrophy, and eventually undergo apoptosis, leaving behind the scaffolding for bone cells to invade and form new bone. In patients with JIA, affected joints can exhibit bony overgrowth and this may occur through EBF. These current findings indicate JFLS display a hypertrophic chondrocyte-like phenotype which is enhanced upon BMP4 stimulation. In the presence of BMP4, not only did FLS undergo hypertrophy with increased COLX, but also secreted bone-derived ALP, a marker typically associated with bone cells and hypertrophic chondrocytes, indicating that FLS may play a key role in the bony overgrowth unique to patients with JIA. Additionally, JFLS can influence chondrocytes to become highly proliferative. Using these cell culture models, we are able to demonstrate that FLS influence Ch to express key components necessary for the process of EBF, possibly contributing to leg length discrepancies in affected joints. Our findings further suggest that regulation of BMP may alter the clinical course of children with JIA.

Although our study demonstrates that JFLS follow a chondrocyte-like lineage and can influence Ch in vitro, there some limitations to working with this cell culture system. Our CFLS cell lines were derived from adults so it must be noted that some of the differences could be age related. Although a lack of pediatric control is limiting, using isolated cell types should reduce the influence of age related factors that would be found in vivo. Additionally, while pooling conditioned media from all three JFLS cell lines could create an issue of generalizability it is less limiting than using a single JFLS cell line derived from synovial fluid from a single patient.

## Conclusion

**Fig. 9:**
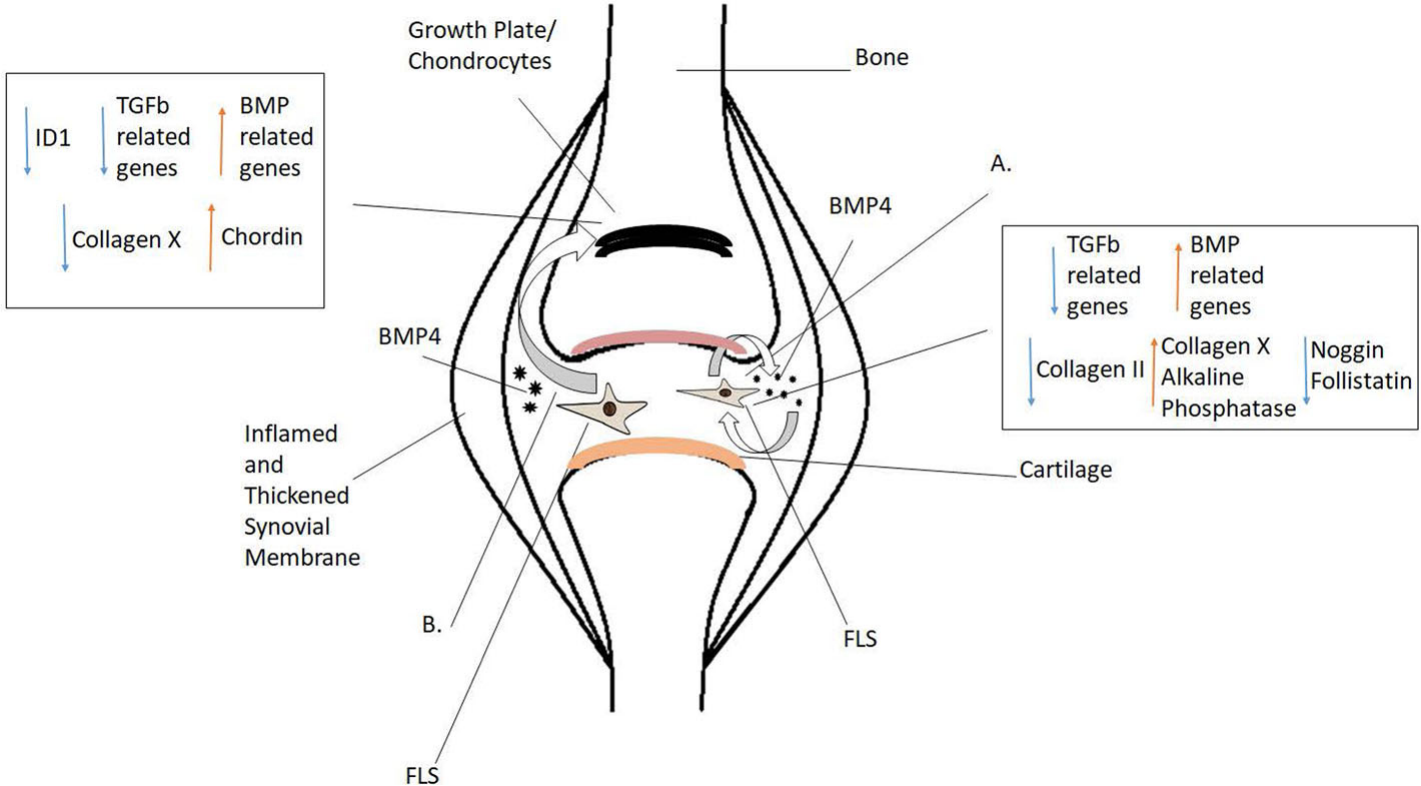
Possible paradigms for the role of FLS in growth disturbances in JIA. JFLS in culture favor expression of genes related to BMP as opposed to TGFβ (**a**). In the presence of exogenous BMP4, JFLS show a decrease in BMP antagonists that have a high affinity for BMP4 which allows for these cells to differentiate further along chondrocyte lineage as demonstrated by an increase in COLX and ALP, late-stage markers, and a decrease in COL2, an early-stage marker (**a**). Cell hypertrophy is required for endochondral bone formation. Similar to JFLS in culture, chondrocytes cultured in JFLS-conditioned media favor expression of genes related to BMP as opposed to TGFβ (**b**). Unlike JFLS in culture, Ch-JFLS decrease protein expression of hypertrophic marker, COLX and have increased protein expression of BMP antagonist, chordin which can contribute to the inhibition of endochondral bone formation (**b**)

Exogenous BMP4 causes FLS to undergo hypertrophy and become less proliferative (Fig. 9a). Given the relevance of BMP signaling in the process of EBF and that increased BMP signaling promotes chondrocyte proliferation while loss of TGFβ induces hypertrophy [47, 48], we studied the interaction of FLS with already differentiated chondrocytes in vitro. Chondrocytes cultured in JFLS-conditioned media had a significant decrease in COLX, and favored expression of chordin, a BMP antagonist that can inhibit endochondral bone formation (Fig. 9b). FLS influenced chondrocytes to remain in the proliferating stage of chondrocyte differentiation and prevented chondrocytes from entering into hypertrophy. Importantly, JFLS appear to have a stronger impact on this phenomenon than CFLS as noted by a significant increase in the chondrocyte proliferation marker COL2 in Ch-JFLS compared to Ch-CFLS. Ch-JFLS demonstrated a greater degree of BMP upregulation compared to Ch-CFLS.

While literature supports that cytokines like TNF-α, IL-1β, and IL-6, in conjunction with growth factors and hormones like IGF-1, PTH, and GH, contribute to growth disturbances within the growth plate and synovitis associated with JIA, this is the first study to suggest that FLS and chondrocytes can play a role in the bony overgrowth [49, 50]. In addition, BMPs and regulation of these growth factors play a key role in the interaction between these two prominent cell types. Thus, it cannot be ignored that these findings are clinically relevant to the pathogenesis of JIA. Further examination through the inhibition of BMP signaling and changes in its regulation may elucidate a possible mechanism for disordered long bone growth in JIA.

## Supplementary Information

**Additional file 1:** **Table 1.** Rank Product Analysis of Ch, CFLS, and JFLS. In order to compare across different cell types, Rank Product Analysis was performed on all 21,448 transcripts included on Clariom S Array. Table includes gene symbol, gene name, and the ranking of genes with a pfp < 0.01. Higher ranks, meaning numbers closer to 1, reflect higher expression levels of that gene while lower ranks reflect decreased gene expression levels of a particular gene.

**Additional file 2:** **Table 2.** TGFβ Superfamily Genes. Compiled list of all 27 genes that were analyzed to determine significant genes related to both TGFβ and BMP signaling. This list contains prominent ligands, receptors, and signal transducing genes that regulate signaling in these pathways.

## Data Availability

The datasets used and/or analyzed during the current study are available from the corresponding author on reasonable request.

## Abbreviations

JIA: Juvenile Idiopathic Arthritis
FLS: Fibroblast-like Synoviocytes
JFLS: JIA FLS
BMP4: Bone Morphogenetic Protein 4
EBF: Endochondral bone formation
Ch: Chondrocytes
CFLS: Control FLS
COL2: Collagen II
COLX: Collagen X
ALP: Bone-derived Alkaline Phosphatase
Ch-CFLS: CFLS-conditioned media
Ch-JFLS: JFLS-conditioned media
