# Supplementary figures and images for: Juvenile idiopathic arthritis fibroblast-like synoviocytes influence chondrocytes to alter BMP antagonist expression demonstrating an interaction between the two prominent cell types involved in endochondral bone formation
Source: Pediatr Rheumatol Online J. 2020 Nov 16;18:89. doi: 10.1186/s12969-020-00483-0 (PMC7670793; doi:10.1186/s12969-020-00483-0)

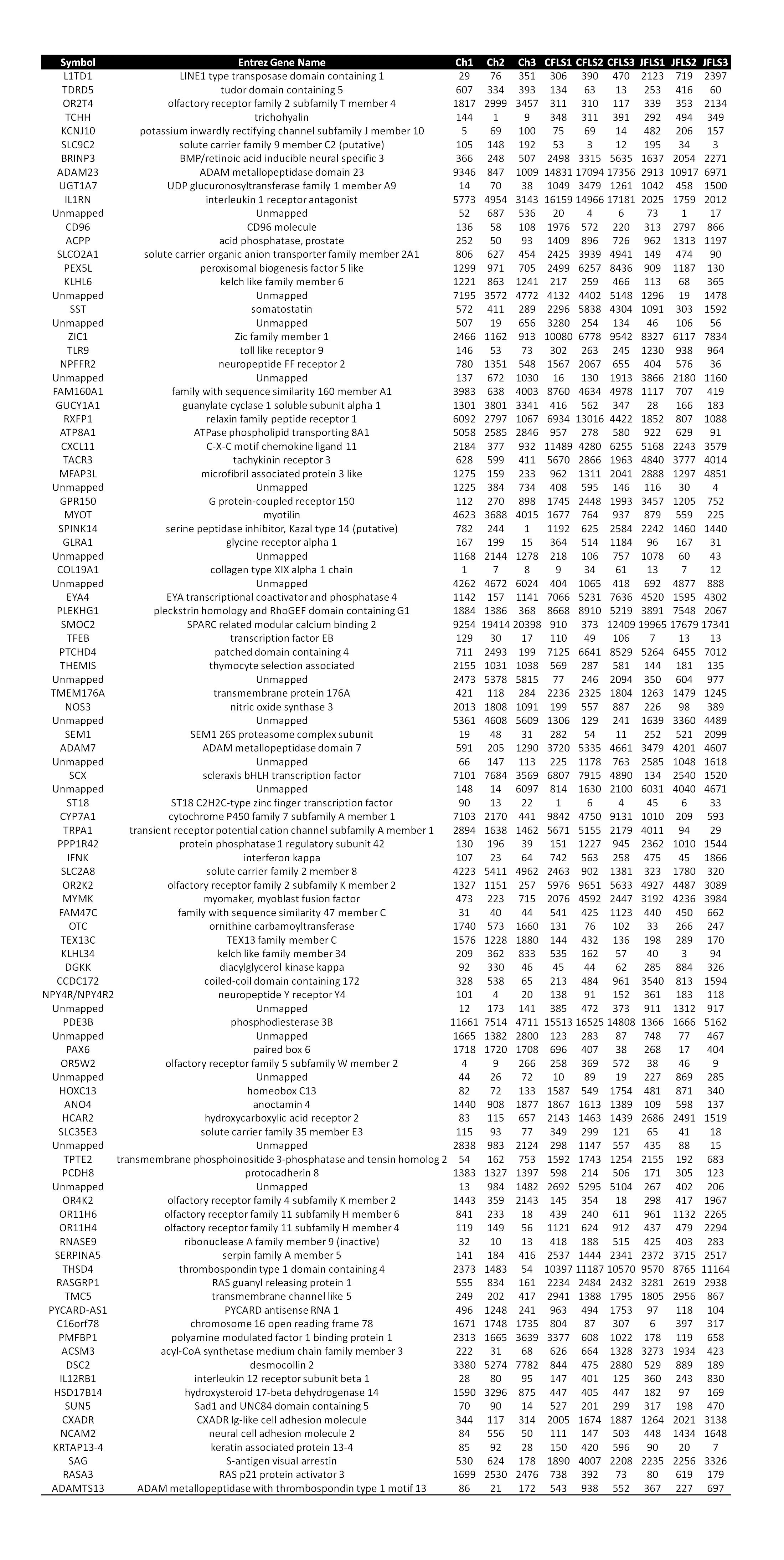

Supplement: Supplementary file 1 — Additional file 1: Table 1. Rank Product Analysis of Ch, CFLS, and JFLS. In order to compare across different cell types, Rank Product Analysis was performed on all 21,448 transcripts included on Clariom S Array. Table includes gene symbol, gene name, and the ranking of genes with a pfp < 0.01. Higher ranks, meaning numbers closer to 1, reflect higher expression levels of that gene while lower ranks reflect decreased gene expression levels of a particular gene. [file 12969_2020_483_MOESM1_ESM.tif]

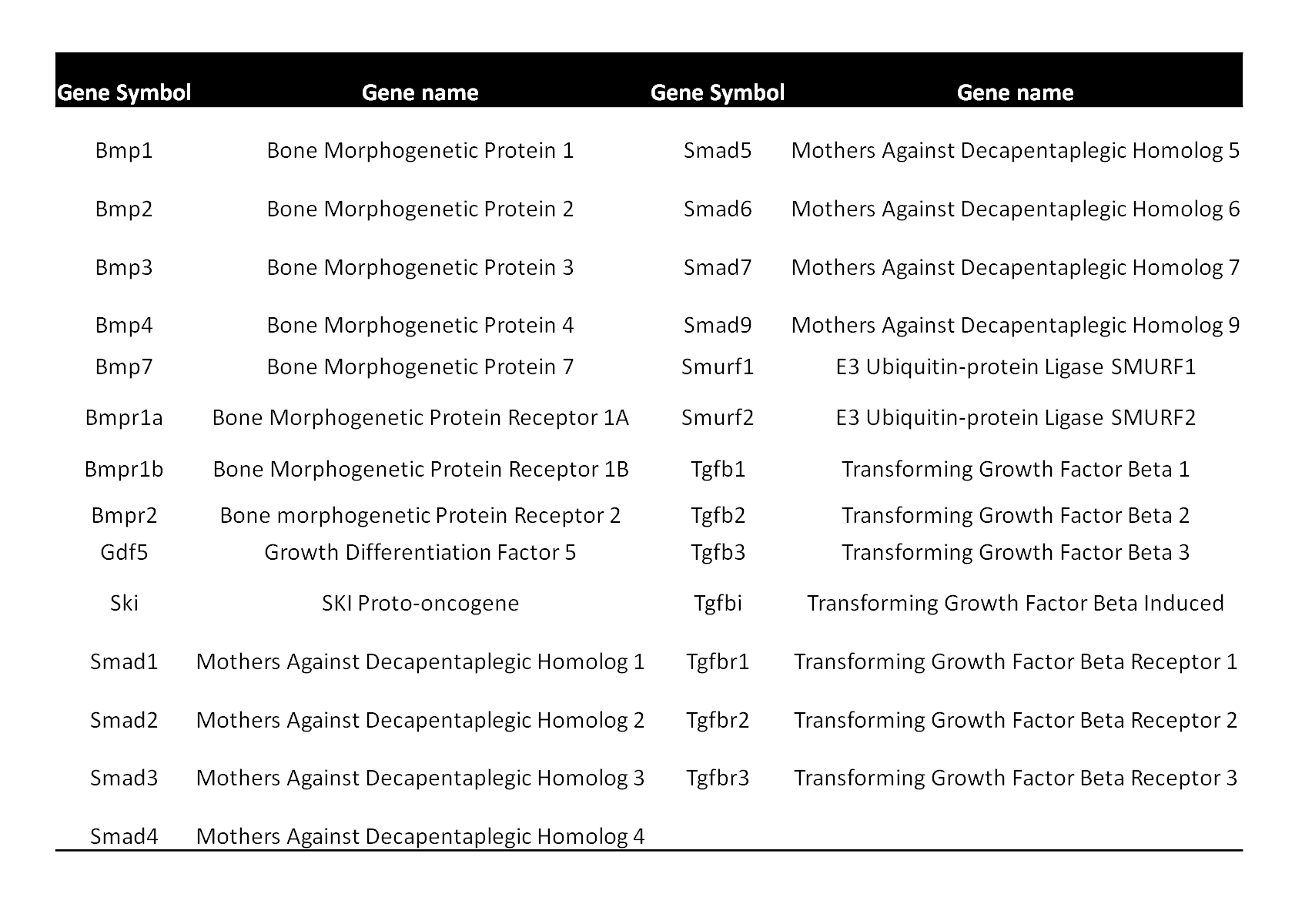

Supplement: Supplementary file 2 — Additional file 2: Table 2. TGFβ Superfamily Genes. Compiled list of all 27 genes that were analyzed to determine significant genes related to both TGFβ and BMP signaling. This list contains prominent ligands, receptors, and signal transducing genes that regulate signaling in these pathways. [file 12969_2020_483_MOESM2_ESM.tif]
